# Supplementary figures and images for: ves1α genes expression is the major determinant of Babesia bovis-infected erythrocytes cytoadhesion to endothelial cells
Source: PLoS Pathog. 2025 Apr 28;21(4):e1012583. doi: 10.1371/journal.ppat.1012583 (PMC12064010; doi:10.1371/journal.ppat.1012583)

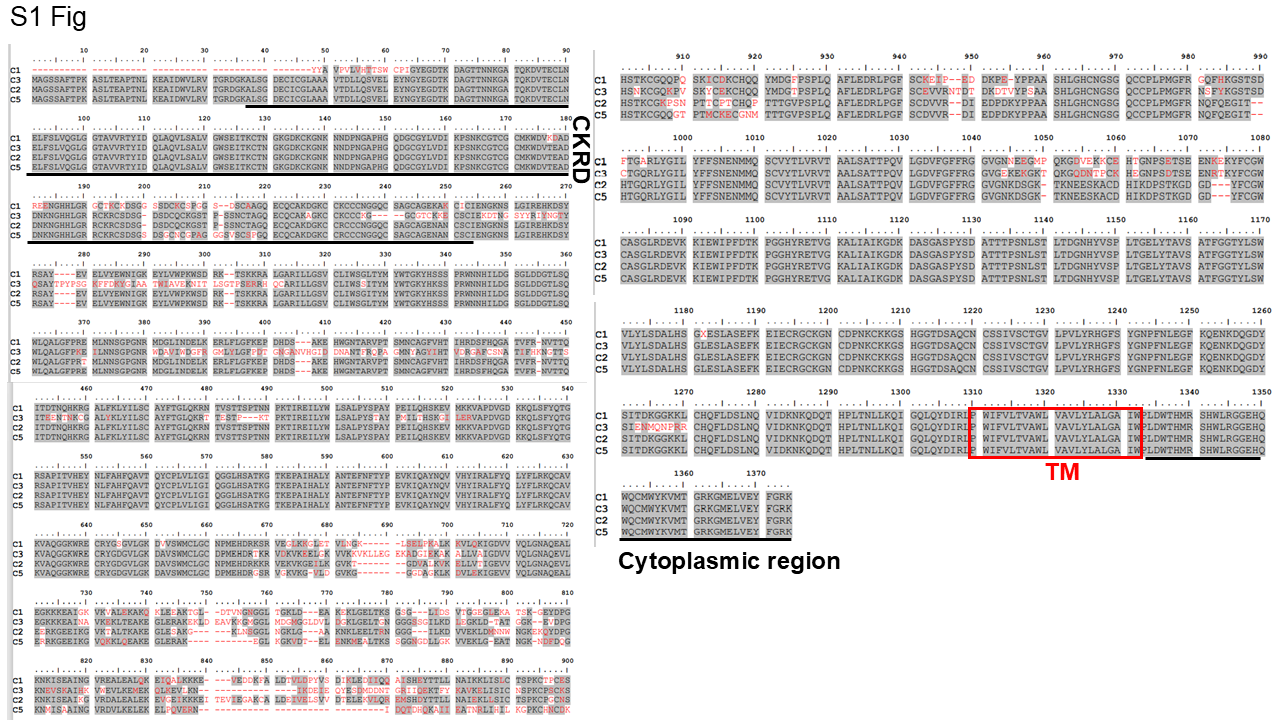

Supplement: S1 Fig — Multiple amino acid sequence alignment of VESA1a using Clustal Omega. Comparison of VESA1a sequence revealed by RNA-seq and primer walking among cytoadherent clones. Cysteine and lysine-rich domain (CKRD) and cytoplasmic region are underlined. The transmembrane domain is (TM) boxed. (TIFF) [file ppat.1012583.s001.TIF]

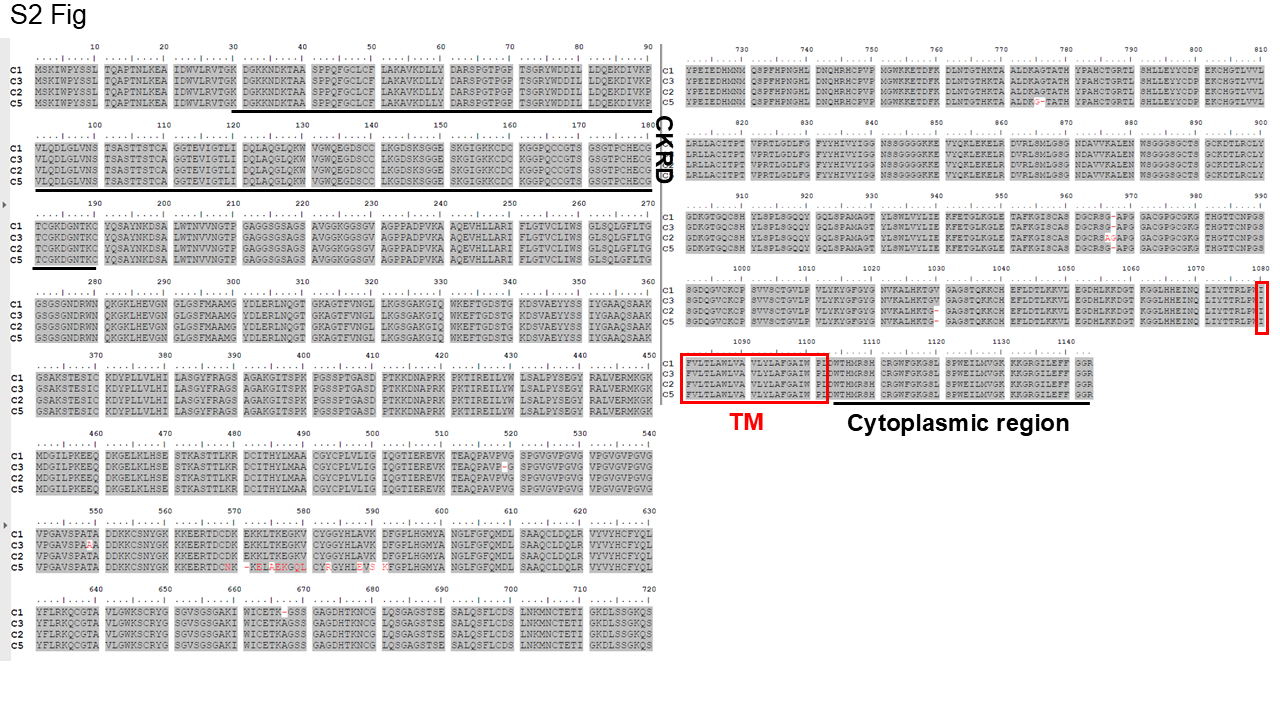

Supplement: S2 Fig — Multiple amino acid sequence alignment of VESA1b using Clustal Omega. Comparison of VESA1b sequence revealed by RNA-seq among cytoadherent clones. Cysteine and lysine-rich domain (CKRD) and cytoplasmic region are underlined. The transmembrane domain is (TM) boxed. (TIFF) [file ppat.1012583.s002.TIF]

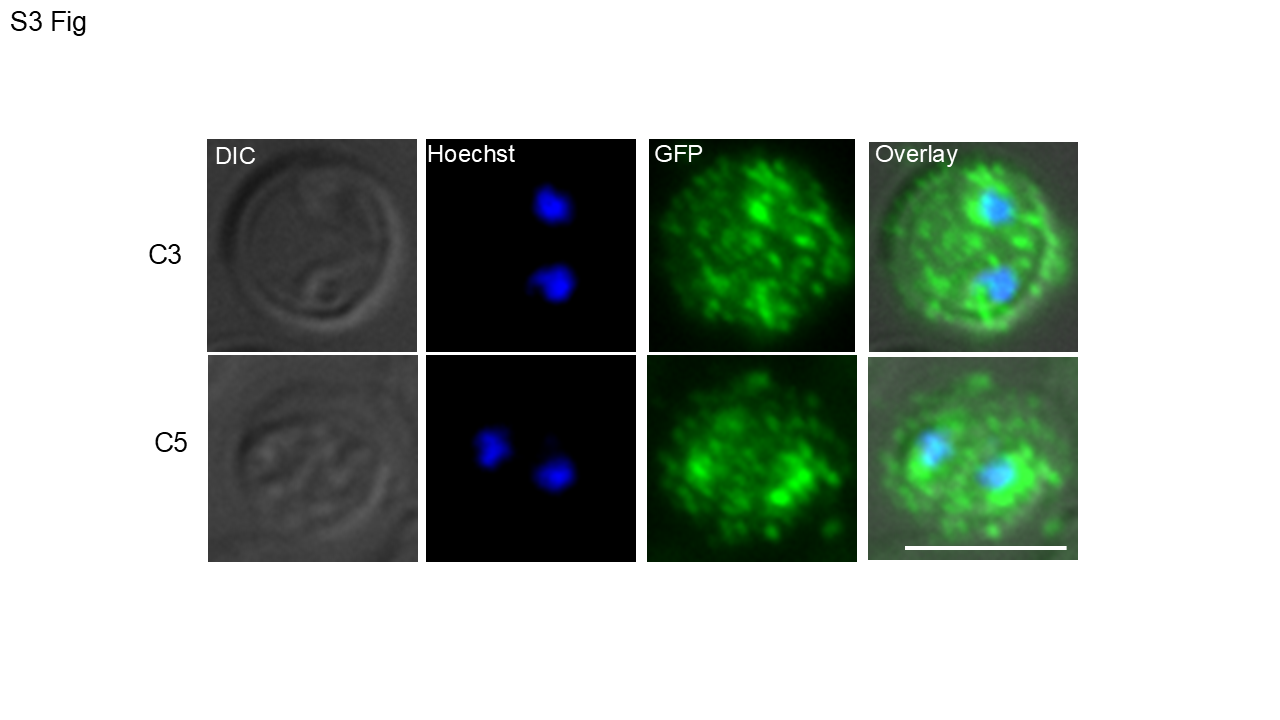

Supplement: S3 Fig — Immunofluorescence microscopy of transgenic parasites episomally expressing VESA1a-GFP. The parasites were reacted with anti-GFP antibody (green) and nuclei were stained with Hoechst 33342 (Hoechst, blue). All the signals were taken at the same focal plane. Scale bar = 5 μm. (TIFF) [file ppat.1012583.s003.TIF]

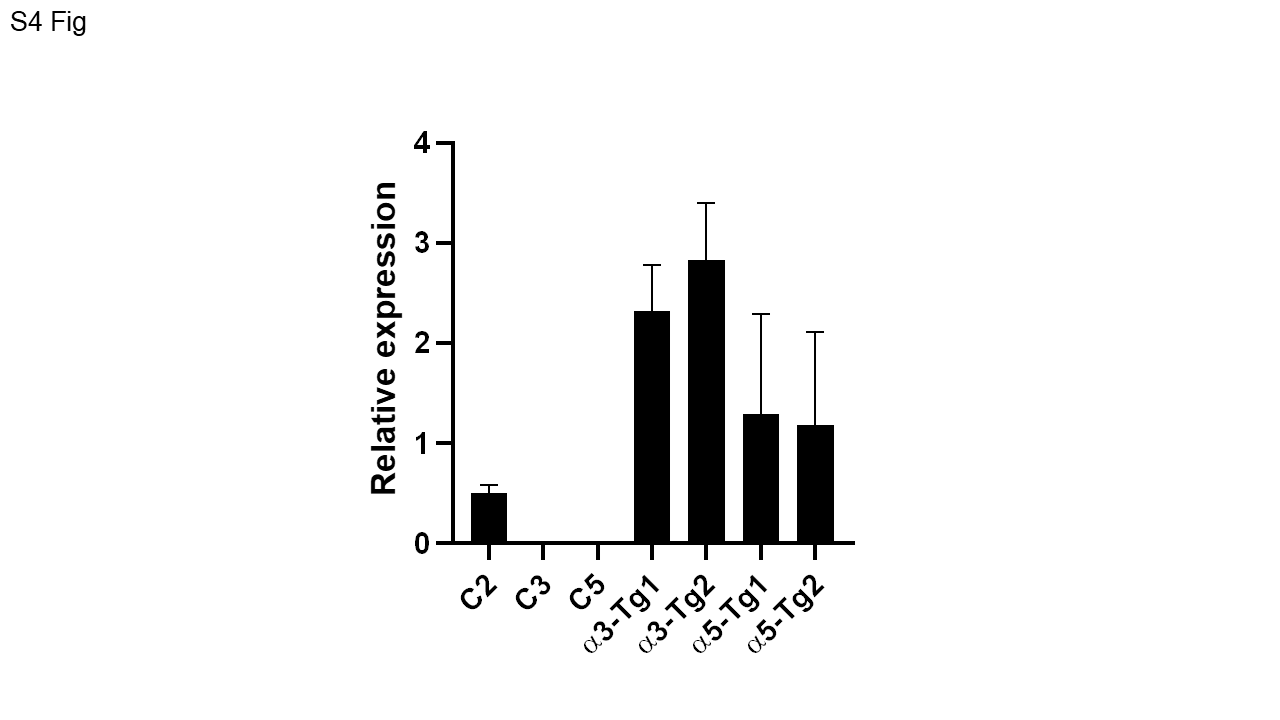

Supplement: S4 Fig — Quantitative reverse transcription PCR (qRT-PCR) of ves1α. Relative transcript levels of C2 type ves1α in C2, C3, C5, and transgenic parasites were quantified using qRT-PCR. α3-Tg1 and Tg2: transfectants expressing C3 ves1α on C2 clone parasite; α5-Tg1 and Tg2: transfectants expressing C5 ves1α on C2 clone parasite. All data are shown as mean ± SD. Transcript levels were normalized against methionyl-tRNA synthetase (Gene ID: BBOV_I001970). (TIFF) [file ppat.1012583.s004.TIF]
