# Supplementary material for: ves1α genes expression is the major determinant of Babesia bovis-infected erythrocytes cytoadhesion to endothelial cells
Source: PLoS Pathog. 2025 Apr 28;21(4):e1012583. doi: 10.1371/journal.ppat.1012583 (PMC12064010; doi:10.1371/journal.ppat.1012583)
Supplement: Attachment — Submitted filename: Reviewer.docx [file ppat.1012583.s009.docx]

**Reviewer’s comments**

The manuscript "Ves1α genes expression is the major determinant of Babesia bovis infected erythrocytes cytoadhesion to endothelial cells" is well-written and presents interesting results. The authors utilized bovine brain endothelial cells to investigate the molecular mechanisms of cytoadhesion of B. bovis. They selected clones with the ability of cytoadhesion using bovine brain endothelial cells. Furthermore, the authors conducted transcriptome analysis of the clones and whole genome sequences.

Please take note of the following concerns regarding the manuscript: This manuscript is based on cloning Babesia parasites to test whether VESA1a is responsible for cytoadhesion. The description of the experiment lacks details important for the conclusion.

1) Cloning a single infected RBC from bovine brain endothelial cells containing multiple attached RBCs is not clearly explained.

2) The authors did not specify the number of passages of the Babesia culture after cloning the parasites.

3) It is unclear why the authors did not select a clone that lacked cytoadhesion ability. This would have allowed them to demonstrate the restoration of cytoadhesion capability by transfected clones.

4) The authors did not provide information on how they restored the RBC culture with the cytoadhesion clones, nor did they specify the parasitemia used for RNA and DNA isolation.

Minor concerns:

Few typos throughout the manuscript.
