# Supplementary material for: ves1α genes expression is the major determinant of Babesia bovis-infected erythrocytes cytoadhesion to endothelial cells
Source: PLoS Pathog. 2025 Apr 28;21(4):e1012583. doi: 10.1371/journal.ppat.1012583 (PMC12064010; doi:10.1371/journal.ppat.1012583)
Supplement: Attachment — Submitted filename: Answer to Reviewer_a.docx [file ppat.1012583.s011.docx]

**Manuscript ID: PPATHOGENS-D-24-01946**

Title: *ves1α* genes expression is the major determinant of *Babesia bovis*-infected erythrocytes cytoadhesion to endothelial cells

Authors: Hassan Hakimi, Junya Yamagishi, Miako Sakaguchi, Atefeh Fathi, Jae Seung Lee, Guilherme G. Verocai, Shin-ichiro Kawazu, Masahito Asada

We would like to express our gratitude to the editors and reviewers for their careful consideration of our manuscript. We have revised the manuscript according to the reviewers’ comments, described below and shown in red font in the revised manuscript. We added two authors (Atefeh Fathi and Jae Seung Lee) that contributed to the manuscript revision by performing some of the experiments.

**Major Issues: Key Experiments Required for Acceptance**

**Reviewer #1: Major concerns**

**1. Gain-of-function experiments. While this is a logical, powerful approach and the centerpiece of the authors’ argument, there are significant flaws in the way this experiment was performed. Some data are also unconvincing. (i) Figure 3B. The results of the western blots are internally inconsistent. Anti-GFP results suggest the presence of VESA1a-GFP fusions of about 160 kDa in a3-Tg1 and 2, and about 180 kDa in a5-Tg1 and 2. In contrast, anti-VESA1 results indicate a single, major VESA1a protein of consistent size (about 150 kDa) in the C2 parental line and each of the selected variant clones. No evidence of the GFP fusion protein is seen despite its presumed overexpression. Please explain.**

We think that the anti-VESA1 peptide antibody is not detecting the chimeric C3 and C5 type VESA1a-GFP protein and the consistent equal band in this Western blot indicates expression of original C2 clone VESA1a in all transgenic parasites. Additionally, IFAT result (S3 Fig) is consistent with the Western blot showing the expressing and export of chimeric VESA1-GFP.

We performed qRT-PCR which confirmed that the *ves1α* from C2 is being expressed in all transgenic lines. Considering these results, we believe that transgenic parasites are expressing two VESA1a, the C2 type VESA1a as C2 clone was used for transfection and the chimeric VESA1a-GFP (S4 Fig, line 323-326, 795-800).

**(ii) Figure 3C. It is unclear why the authors would choose to use the cytoadherent C2 line to perform gain-of-function experiments for the adherent phenotype when this line is already cytoadherent. This choice is illogical, particularly when non-adhesive lines could have been obtained for this use, and may underlie the highly varied results obtained with the various transgenic parasite lines (alternatively, both C2 and a non-adhesive line could be engineered for contrast).**

We started panning the parasites for binding to BBEC with using an uncloned parasite population to have a higher chance of getting cytoadherent parasites. Therefore, we were not able to use a non cytoadherent clonal line for making transfectants. We decided to use C2 as a low cytoadherent line with known genome and transcriptome (done within this study) as the parasite line for the gain-of-function studies.

**(iii) A control transgenic line in which a non-adhesive ves1a gene was similarly expressed should have been included to demonstrate a failure to provide this function. Similarly, ves1b genes could have been used to demonstrate (presumed) failure to impart the cytoadherent phenotype.**

As mentioned above, we started cytoadhesion assay with an uncloned parasite population, thus we do not have *ves1α* sequence of non-cytoadherent parasite before panning.

To validate any potential contribution of VESA1b to cytoadhesion, we episomally transfected C2 with *ves1β*-*mCherry-bsd*-expressing plasmids that express *ves1β* from C3 (high-binding clone) or C5 (low-binding clone) (Fig 4A). Western blot analysis confirmed the expression of the chimeric VESA1b-mCherry in two transgenic lines generated from each plasmid (Fig 4B). We proceeded with these transgenic lines for cytoadhesion assay. No significant increase was seen in binding to BBECs in transgenic lines compared with C2 confirming that VESA1b had no role in cytoadhesion (Fig 4C). (line 210-233, 407-417).

**(iv) The authors present no data confirming that the original C2 ves1a gene is being expressed in the transgenic lines, while attributing all binding to the inserted ves1a genes. Given the parasite’s rapid antigenic variation, transgenic lines may or may not still be expressing the original C2 ves1a gene. This seriously compromises interpretation of the results.**

We performed qRT-PCR which showed that the *ves1α* from C2 is being expressed in all transgenic lines. Therefore, we considered this into interpretation of results and modified the manuscript accordingly (323-326).

**(v) The C2 ves1a itself should also be inserted into the EF1a site to assess the effects of VESA1a/VESA1b stoichiometry on cytoadherence. Without the above experiments I do not believe it is possible to make rigorous interpretations regarding the contributions of either VESA1a or 1b in the cytoadherence function.**

We performed qRT-PCR which confirmed that the *ves1α* from C2 is being expressed in all transgenic lines. Considering this finding we added this statement in the discussion:

We cannot rule out the possibility that VESA1a expression level may contribute to cytoadhesion ability as all transgenic parasite lines express C2 type VESA1a (S4 Fig). A better genetic tool for controlled expression of *ves1α* is needed to test this hypothesis (line 323-326).

**(vi) The inserted ves1a genes are likely transcribed at high levels throughout the cell cycle because of the highly active EF1a promoter. VESA1a protein may have been expressed at times and/or levels that are not reflective of the natural situation, requiring caution in interpreting the results.**

We added this statement in the discussion section:

Given that *ves1α* in transgenic lines are driven by the strong promoter of *ef-1α*, VESA1a protein may have been expressed at times and/or levels that are not reflective of the natural situation (line 305-307).

**2. line 150. An alternative and potentially more plausible interpretation is that VESA1b is responsible for the cytoadherence phenotype, since selection resulted in the expression and/or maintenance of essentially the same form of the protein, whereas significant changes in the sequence of VESA1a did not have a qualitative effect. Unfortunately, the authors did not provide pre-selection sequences or sequences from non-cytoadherent lines to know what was already being transcribed or associated with non-adhesive phenotype, nor was this possibility addressed in the gain-of-function experiments. It is interesting to note from Figure 2 that the stoichiometry of ves1a/ves1b transcripts is much higher in the C1 and C3 high-adhesion lines than in the C2 and C5 low-adhesion lines. Stoichiometry of the two subunits may be significant to expression of the adhesive function.**

As explained in response to comment 1-(iii), we episomally transfected C2 with *ves1β*-*mCherry-bsd*-expressing plasmids that express *ves1β* from C3 (high-binding clone) or C5 (low-binding clone) (Fig 4A). Western blot analysis confirmed the expression of the chimeric VESA1b-mCherry in two transgenic lines generated from each plasmid (Fig 4B). We proceeded with these transgenic lines for cytoadhesion assay. No significant increase was seen in binding to BBECs in transgenic lines compared with C2 confirming that VESA1b had no role in cytoadhesion (Fig 4C). (line 210-233, 407-417).

Regarding the stoichiometry of *ves1α/ves1β,* as shown in Fig 2, this value is higher in C1 and C3 than C2 and C5. Thus, we cannot rule out the possibility that VESA1a expression level may contribute to cytoadhesion ability as all transgenic parasite lines express C2 type VESA1a (S4 Fig). We added this statement in line 323-326. However, we believe that our conclusion on “VESA1a is the determinant of *B. bovis* cytoadhesion” is consistent with the data provided in this manuscript.

**3. Figure 3C. The mapping of reads to the sequences included in the figure is sometimes not convincing. How confident are the authors that they have identified the correct expression site? Some, such as the “blue” C5 ves1a sequences appear as though a short sequence may have come from that site but also be a part of a different, complete, transcribed gene. This is a pattern one might see when some sequences in the transcribed gene are derived by segmental gene conversion. This affects interpretation of LAT identity, monoallelic exclusion, etc.**

We agree with the reviewer’s concern. Mapping of *ves1* reads over the genome is challenging as they share high homology. Thus, we performed whole genome sequencing using long-reader MinION and aligned the RNA-seq reads on the corresponding parasite clone genome to mitigate miss assembly. This strategy was successful in finding LAT for C1, C2, and C3. However, we could not find a major LAT for C5 which could be due to miss assembly or lack of clear LAT in this parasite clone. We mentioned all these limitation in the results section (line 256-267).

**Reviewer #2:**

**Three major concerns dampen overall enthusiasm.**

**1) Lines 143 and 144 state that “Comparison of transcripts between 2 high and 2 low cytoadherent clones did not reveal any significant hits (Sup Table 1).”, yet I see eight genes with expression upregulated and nine genes with expression downregulated with increased binding (all with p-values <0.05 – some with p-values as low as 10^-16) in this Table. It is unclear why these genes are not discussed and instead the ves genes are the focus of investigation.**

None of the proteins encoded by the genes listed in S1 Table have classical ligand structure (transmembrane domain and extracytoplasmic region) or are exported proteins (having signal peptide), thus we think they are not candidate ligand for cytoadhesion. We corrected this sentence in the manuscript (line 143-145)

**2) Although briefly discussed, the concern of binding affinity to BBEC being merely a matter of expression level (of any ves1a variant) rather than expression of certain ves1a variants needs to be further discussed. The expression levels of the variants shown in Figure 2A reveal that the two ‘high binders’ have the higher levels of expression than the two ‘low binders’. Concern regarding levels of surface ves1a protein expression being directly related to binding affinity is also raised in the transfection experiments when the transfection of ‘low binder’ ves1a into a low binding construct also results in increased binding (although not significant). To combat this criticism, RT-PCR determined expression levels of the ves1a constructs in Fig 3C could be measured. This could determine whether the alternate binding affinities are indeed due to alternate construct sequences or merely to overexpression of any vas1a construct. An interesting control would also be to transfect the C2 construct into itself – under the ef-1a promoter. This would dissect out the roles of the sequence and the expression level of a particular ves1a sequence.**

We performed qRT-PCR which confirmed that the *ves1α* from C2 is being expressed in all transgenic lines. Considering this finding we added a paragraph in the discussion:

We cannot rule out the possibility that VESA1a expression level may contribute to cytoadhesion ability as all transgenic parasite lines express C2 type VESA1a (S4 Fig). A better genetic tool for controlled expression of *ves1α* is needed to test this hypothesis (line 323-326).

**3) The mapping of the LAT does not seem to be consistent. The text states that it is on Ch2 on two of the clones, in multiple sites for one and undetermined in the fourth. However, this text does not agree with the designation of LATs (by blue and red triangles) in Figure 4B, where there are LATs discovered in each of the clones. A discussion as to how this divergence could have happened so quickly (21 rounds of selection) is warranted. As well, an overall discussion as to why the location of LATs is of scientific interest would be helpful**.

We corrected this discrepancy in Fig 5 and figure legend (line 280-281)

We started panning the parasites for binding to BBEC using an uncloned parasite population to have a higher chance of getting cytoadherent parasites. RNA-seq indicated the expression of different variants of VESA1a in 4 clones which likely resulted from genomic recombination, or these variants were selected by panning (S1 Fig) (line 145-148).

Regarding the scientific interest of finding LAT, we wanted to know whether there is a specific location in genome of *B. bovis* that function as LAT or the LAT position changes across 4 chromosomes of *B. bovis*. Finding LAT location will facilitate understanding antigenic variation of *B. bovis* and will be a foundation for developing novel control strategies targeting antigenic variation (line 349-351).

**Reviewer #3:**

**Please take note of the following concerns regarding the manuscript: This manuscript is based on cloning Babesia parasites to test whether VESA1a is responsible for cytoadhesion. The description of the experiment lacks details important for the conclusion.**

**1) Cloning a single infected RBC from bovine brain endothelial cells containing multiple attached RBCs is not clearly explained.**

The panning of parasite and cloning of cytoadherent parasite line was added to the Methods section.

Panning the parasites for binding to bovine brain endothelial cells (BBECs; Cell Applications Inc., USA) and cytoadhesion assays were performed as described [21]. Briefly, BBECs were seeded in 6 well plates in the growth medium. Following 2-3 days of culture when the cells became confluent, *B. bovis*-iRBCs with 2–5% parasitemia and 1% hematocrit were added to the BBEC culture. The cells were incubated for 90 min with gentle agitation every 15 min. Nonadherent iRBCs were removed by washing with Hanks-balanced salt solution. Fresh uninfected RBCs were added to each well and incubated overnight. The RBC suspension was removed and transferred to a new plate and cultured under normal condition. Once parasitemia reached 2-5%, a new round of selection was performed. The cytoadherent parasite line from the 21^st^ round of panning was cloned by limiting dilution (line 439-450).

**2) The authors did not specify the number of passages of the *Babesia* culture after cloning the parasites.**

Following cloning we cryopreserved several stocks from each clones as described (392-394).

The parasites were maintained in the culture no more than 5 passages to minimize the chance of *ves1* recombination and switching (line 456-458).

**3) It is unclear why the authors did not select a clone that lacked cytoadhesion ability. This would have allowed them to demonstrate the restoration of cytoadhesion capability by transfected clones.**

We started panning the parasites for binding to BBEC using an uncloned parasite population to have a higher chance of getting cytoadherent parasites. Therefore, we were not able to use a non cytoadherent clonal line for making transfectants.

**4) The authors did not provide information on how they restored the RBC culture with the cytoadhesion clones, nor did they specify the parasitemia used for RNA and DNA isolation.**

The detailed protocol added to the Methods section:

*In vitro* cultured parasites were cryopreserved in 20% polyvinylpyrrolidone 40K (w/v) (P0930, Sigma-Aldrich) in the Vega and Martinez solution as described [42]. (line 392-394).

Genomic DNA was extracted from C1, C2, C3 and C5 *in vitro* cultured clones with parasitemia of 5-10% using NucleoSpin Blood QuickPure kit (Macherey-Nagel GmbH, Düren, Germany) (line 460-462).

**Part III – Minor Issues: Editorial and Data Presentation Modifications**

**Reviewer #1: Minor points**

**1. line 103. Many ves gene clusters are internal, not subtelomeric, distinguishing this parasite from Plasmodium, Trypanosoma, and many others.**

This sentence is corrected (line 102-104).

**2. line 136. Perhaps the authors could comment on why the Texas strain required 16 rounds of panning to detect cytoadherent parasites. As I recall, in the original description of the method (O’Connor et al. 1999. Infect. Immun.) those authors were able to recover cytoadherent parasites with only a few selections. Is the difference in the parasites or methodology? Please comment.**

The parasite line that was used in this study has been in the *in vitro* culture for more than 20 years in our institute and was not passaged in cattle or selected for binding to BBECs. This could be the reason why 16 rounds of selection were needed to get the cytoadherent line.

**3. line 145. Because of their prior association with expression of the cytoadherence phenotype the authors should include information on transcript levels of the genes encoding SBP2t11 and VEAP proteins (Gallego-Lopez et al. 2018. Intl. J. Parasitol.; Hakimi et al. 2020. PLoS Pathogens), and perhaps comment on their apparent expression stability.**

Among other candidates that previously shown to participate in cytoadhesion [22-24], the expression of *veap* and *sbp3* was not significantly different between high-binding and low-binding clones (S2 Table). Additionally, *sbp2t11* was not expressed in any of these clones (line 151-154).

**4. Figure 3A. Please identify the “2A” element and the locations of (relevant) stop codons. In Figure 3C please add a significance bar comparing C2 and C5.**

In the construct, *gfp* and *hdhfr* are in frame with *ves1α* and stop codon located after *hdhfr* (added to Fig 3A). C2 and C5 binding was not significantly different. The significance bar was added to Fig 3C.

**5. line 206. As worded, it sounds as though ves1a and ves1b are mutually exclusive in their expression. Please rephrase to make it clear that different ves1a are mutually exclusive from other ves1a, and ves1b from other ves1b.**

This sentence is corrected (line 236-238).

**6. line 319. It is confusing that the ves1b sequences were conserved, yet variant ves1a transcripts were arising from different expression sites. Are the bidirectional “LAT”s transcribed in only one direction?**

As shown in Fig 5C, simultaneous expression of *ves1α* and *ves1β* genes in LAT suggests existence of a bidirectional promoter in the intergenic region as was confirmed previously using firefly luciferase assay (Wang et al., 2012).

**7. line 354. Please indicate whether the cDNAs cloned for use in transgenesis were sequenced to confirm their representation of the population.**

The final plasmid constructs were sequenced by Sanger sequencing to confirm the elements are in frame and the cloned *ves1α* and *ves1β* are from the respective clones (line 415-417).

**8. line 700 (Figure S1). Please move the black bars indicating the CKRD to beneath the sequences so that they are underlining as stated. Currently they are above, indicating incorrect sequences.**

We corrected the bar in S1 Fig.

**9. Figure 1B. It might be helpful to describe for the reader what they are seeing. Without a frame of reference such an image can be confusing.**

We corrected Fig 1B and C legend (line 160-164).

**10. Figures 4A and 4B. Please orient the chromosomal sequences the same way in the dot plots and synteny plots (from Chr 1 to 4 or Chr 4 to 1 left to right in each, not both orientations). This will aid the reader in interpretation.**

We corrected the order of chromosomes in Fig 5A and B.

**11. Figure 5. Please include an in-figure index of the colors to identify what each pie wedge represents.**

Considering frequent recombination in *ves1* containing regions in the genome, the gene IDs from T2Bo reference strain (which is based on the location of genes in the genome) does not show the actual location of *ves1α* and *ves1β* in our parasites. However, we added the IDs from the LATs or major expressing loci in Fig 6.

**12. Figure 6. Please indicate whether the Hoechst and GFP images were obtained as single exposures at the same focal plane or are compiled z-stacks. This affects interpretation of the location of the VESA1a-GFP polypeptides within the parasite and erythrocyte, and thus its availability to mediate cytoadherence. It would be helpful if the images could be provided at the same magnification.**

All the signals were taken at the same focal plane (line 793-794) and the images are provided at the same magnification.

**Reviewer #2: Other more minor areas of concern:**

**Line 53 – I am not sure that the data speak at all to cytoadherence specificity. Please elaborate. I see no data discussing potential receptor ligands. That is what I think of when I think of specificity. Is something else meant by this phrase here?**

We corrected this sentence (line 53-55).

**Line 291 – The discussion of endothelial receptors for PfEMP1 interactions is inadequate. At a minimum EPCR binders should be mentioned.**

Cellular adhesion of *P. falciparum*-iRBCs consists of a rolling interaction with ICAM1 and a stationary interaction with CD36 and EPCR on vascular endothelial cells which leads to severe malaria [28-30]. (line 330-332).

**Line 293 – The authors state that: ‘using a static cytoadhesion assay, we were not able to find novel ligands for binding of iRBCs ‘ , yet is does not seem that this was one of the aims of the paper. Did they attempt to look for other ligands and fail? If so, these data are not shown in the current manuscript.**

The original aim of this study was to find novel genes or ligands responsible for cytoadhesion. To find the genes responsible for cytoadhesion, 4 clones of cytoadherent *B. bovis*, 2 high-binding (C1 and C3) and 2 low-binding (C2 and C5), were subjected to RNA-seq. We considered up- or downregulated genes with more than two-fold change in the expression and a significant *P*-value (P≤0.05). Comparison of transcripts between 2 high and 2 low cytoadherent clones did not reveal any significant hits that have ligand structure or export motif (S1 Table) (Line 140-145)

**Figure 5 – clone 5: There is no beta chain labeled in the pie chart and two alphas. Is that correct? Or are one of those supposed to be a beta?**

Yes, we could not find any major expressing *ves1β* in C5.

**Reviewer #3: Few typos throughout the manuscript**

The typos throughout the manuscript are corrected.
